# Sexually dimorphic characteristics of dopamine D1 receptor-expressing neurons within the shell of the nucleus accumbens of adolescent mice

**DOI:** 10.21203/rs.3.rs-3717874/v1

**Published:** 2023-12-16

**Authors:** Heather C Aziz, Regina A Mangieri

**Affiliations:** The University of Texas at Austin College of Pharmacy; The University of Texas at Austin College of Pharmacy

**Keywords:** Glutamate, Electrophysiology, Synapse, Excitability, Sex Differences, Striatum

## Abstract

**Background:**

Adolescence, a developmental stage, is characterized by psychosocial and biological changes. The nucleus accumbens (NAc), a striatal brain region composed of the core (NAcC) and shell (NAcSh), has been linked to risk-taking behavior and implicated in reward seeking and evaluation. Most neurons in the NAc are medium spiny neurons (MSNs) that express dopamine D1 receptors (D1R+) and/or dopamine D2 receptors (D2R+). Changes in dopaminergic and glutamatergic systems occur during adolescence and converge in the NAc. While there are previous investigations into sex differences in membrane excitability and synaptic glutamate transmission in both subdivisions of the NAc, to our knowledge, none have specified NAcSh D1R+MSNs from mice during mid-adolescence.

**Methods:**

Sagittal brain slices containing the NAc were prepared from B6.Cg-Tg(Drd1a-tdTomato)6Calak/J mice of both sexes from postnatal days 35–47. Stained smears were made from vaginal samples from female mice to identify the stage of Estrous at death. Whole-cell electrophysiology recordings were collected from NAcSh D1R+MSNs in the form of membrane-voltage responses to current injections and spontaneous excitatory postsynaptic currents (sEPSCs).

**Results:**

The action potential duration was longer in males than infemales. Additionally, the frequency of sEPSCs was higher in females, and the mean event amplitude was smaller than that in males. We found no evidence of the observed sex differences being driven by the stage of the Estrous cycle and no physiological parameter significantly varied with respect to the Estrous cycle.

**Conclusions:**

Taken together, our results indicate that NAcSh D1R+MSNs exhibit sex differences during mid-adolescence that are independent of the stage of Estrous, in both AP waveform and glutamate transmission, possibly due to changes in voltage-gated potassium channels and α-hydroxy-5-methyl-4-isoxazolepropionic acid (AMPA) glutamate receptors, respectively.

## Plain Language Summary

Adolescence marks a period of substantial changes in both the mind and body, where alterations in the brain’s structure can influence behavior. One change in behavior exhibited by many adolescents is an increased tendency to take risks, particularly in males. While taking risks can result in positive outcomes, like learning new skills, it can also lead to reckless behaviors that may result in negative outcomes. The nucleus accumbens, a brain region tied to risk-taking and reward perception, is not well-studied during adolescence, particularly in terms of sex differences. Furthermore, puberty occurs during this developmental stage and knowledge of the effects of circulating sex hormones on brain function is limited. To fill these gaps in understanding, this study examined a specific type of brain cell in the nucleus accumbens of adolescent male and female mice. We measured the electrical properties of these cells and assessed how they responded to manipulation of their electrical state. We also measured how much and how often excitatory electrical information is sent to these cells from other brain regions. Our results suggest that these brain cells in males may take longer to communicate information to other brain regions than in females. We also found that, in females, these brain cells received excitatory information more frequently than in males but in smaller amounts. We found no evidence that female hormonal cyclicity affected any parameter or gave rise to the observed sex differences. Understanding these intricacies of brain cell communication sheds light on potential sex-specific vulnerabilities during adolescence.

## Introduction

Adolescence is a stage of development characterized by psychosocial and biological changes that can contribute to an increased propensity to take risks.(1, 2) This tendency toward risk-taking, or choosing an action for which the outcome is more variable, is greater during puberty than childhood and increases across adolescence.(3, 4) During development, risk-taking plays an important role in one’s ability to define oneself and learn new skills, but it can also lead to reckless behavior.(1, 5) Across the span of adolescence, developmental alterations in the brain are widespread, multifaceted, and work to shape the childhood brain into that of adulthood.

One brain structure that is heavily modified during adolescence and linked to risk-taking behavior is the nucleus accumbens (NAc), found within the ventral striatum. Implicated in the seeking and evaluation of reward,(6, 7) the NAc has two subregions: the shell (NAcSh) and the core (NAcC). Both subregions contain dopamine (DA) receptor and glutamate receptor-expressing, -aminobutyric acid-producing (GABAergic) medium spiny neurons (MSNs). NAcSh MSNs project to brain structures mediating decision making, rewarding properties of substances, and reinforcing properties of novelty, and NAcC MSNs innervate structures associated with impulsive choices, spatial learning, and responses to motivational stimuli.(8–10) MSNs have been classified by the subtype of DA receptor that they express: dopamine D1 receptor-expressing (D1R+) or dopamine D2 receptor-expressing (D2R+) MSNs. Both subtypes receive synaptic excitation via glutamatergic inputs from several forebrain structures, and dopaminergic (DAergic) inputs from the midbrain serve to modulate MSN membrane properties and excitability.(9, 11)

During adolescence, drastic changes in both the DAergic and glutamatergic systems occur,(12, 13) some of which appear to be sex specific. Striatal increases in DA concentration, transporters, receptor binding, and receptor expression have been documented as occurring during adolescence.(1, 14–17) Overall, the ratio of D1R + to D2R + neurons in the ventral striatum increases during adolescence, with female mice exhibiting a heightened ratio compared to males.(18) Male D1R expression and binding peaks during adolescence, whereas female D1R expression appears to peak prior to adolescence, and although striatal D1R pruning occurs in both sexes, it occurs at different ages and via different mechanisms in males and females. (19, 20) Regarding glutamate, both the frequency and amplitude of spontaneous excitatory postsynaptic currents in NAc MSNs of male rats were shown to decline during adolescence.(21) Therefore, it is likely that both DAergic and glutamatergic actions in the NAc play a role in the transition from childhood to adulthood.

Puberty, or attaining sexual maturation, occurs during adolescence, is initiated by the brain and is accompanied by increases in gonadal hormones.(1) The terms adolescence and puberty are not synonymous, and the age at which each occurs varies by species, strain, sex, and individual. In rodents, adolescence is considered to occur between postnatal day (PND) 30 and PND 49.(22) In female mice, puberty and first estrus occur between 10–20 days after vaginal opening on PND 26, depending upon the strain.(23, 24) For male mice, puberty is marked by motile sperm, which occurs between PND 40 and PND 55.(25, 26)

Although previous bodies of work have investigated whether MSNs in the NAc exhibit sex-related differences during adolescence and possible effects of the Estrous cycle,(27–29) none of these studies addressed pubertal age directly or specifically evaluated D1R + MSNs in the NAcSh of mice. Considering the evidence for DAergic and glutamatergic changes in the striatum during adolescence, that DA modulates MSN excitability, and that glutamate functions as a major excitatory neurotransmitter in the brain, we examined membrane excitability and synaptic glutamate transmission in NAcSh D1R + MSNs from mid-adolescent mice of pubertal age, gonads intact, to bridge this gap in knowledge.

## Methods

### Subjects

**Figure 1:**
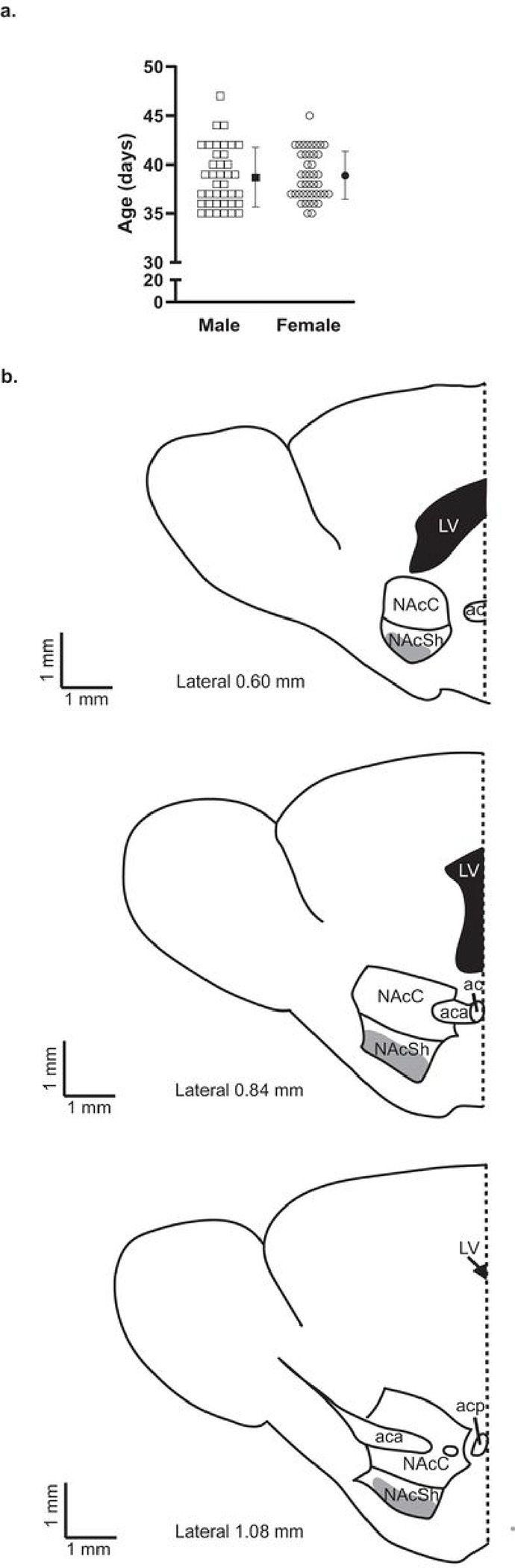
Age on experimental day and representative illustrations of brain slices collected for electrophysiology recordings. ***a.*** Age of male (open squares; n = 39) mice and female (open circles; n = 43) mice on the day of the experiment. Individual values for each mouse (open symbols) are plotted and presented alongside the group mean (closed symbols) and standard deviation. ***b.*** Representative illustrations of the NAc within the sagittal brain sections used for experiments. Gray shading indicates the area from which cells were selected for recordings. The dotted line indicates Bregma. Abbreviations: anterior commissure (ac), anterior part of the anterior commissure (aca), posterior part of the anterior commissure (acp), lateral ventricle (LV), nucleus accumbens core (NAcC), nucleus accumbens shell (NAcSh).

All whole-cell electrophysiology recordings were conducted in brain slices prepared from male and female bacterial artificial chromosome transgenic B6.Cg-Tg(Drd1a-tdTomato)6Calak/J mice (developed by Ade and colleagues (30) (RRID:IMSR_JAX:016204)) from a colony maintained in our laboratory. Animals were group-housed on a 12:12 reverse light cycle (ZT0 = 21:30) in standard mouse cages containing wood chip bedding (Sani-Chips; PJ Murphy). Animals were provided with enrichment in the form of compressed cotton fiber squares (Nestlets; Ancare). Food (Prolab^®^5LL2 RMH 1800; LabDiet) and water were supplied ad libitum. On the day of the experiment, the mice ranged in age from PND 35-PND 47, representing mid-adolescence;(31) the mean age of males was PND 39, and the mean age of females was PND 39 (*t*(80) = 0.3195, *p* = 0.7502)^a^ (Fig. 1a).

All animal procedures were performed in accordance with the University of Texas at Austin’s institutional animal care and use committee’s regulations.

### Brain slice preparation

Animals were lightly anesthetized with isoflurane prior to killing via decapitation. Decapitation took place between 07:00 and 13:30 (ZT9.5 and ZT16). Immediately following removal, the brains were rapidly cooled in ice-cold artificial cerebral spinal fluid (ACSF), continuously bubbled with 95% O_2_/5% CO_2_, and composed of (in mM) 210 sucrose, 26.2 NaHCO_3_, 1 NaH_2_PO_4_, 2.5 KCl, 11 glucose, 6 MgSO_4_, and 2.5 CaCl_2_. Using a vibratome (VT-100S; Leica), 240 μM thick sagittal slices containing the nucleus accumbens were made. Each slice was subsequently transferred to a recovery chamber containing ACSF constantly bubbled with 95% O_2_/5% CO_2_ and composed of (in mM): 124 NaCl, 26 NaHCO_3_, 10 dextrose, 4.4 KCl, 1 NaH_2_PO_4_, 2.4 MgSO_4_, and 1.8 CaCl_2_. Slices were incubated at 32°C in the recovery chamber for a minimum of 60 minutes prior to being transferred to the recording chamber.

The recording chamber contained ACSF composed of (in mM): 124 NaCl, 26 NaHCO_3_, 1 dextrose, 4.4 KCl, 1 NaH_2_PO_3_, 1.2 MgSO_4_, and 2.0 CaCl_2_. To block -aminobutyric acid subtype A (GABA_A_) receptor-mediated currents, recording ACSF also included 50 μM picrotoxin. ACSF was maintained at a temperature ranging from 32°C to 33°C and pumped continuously into the recording chamber at a rate of ~ 2 mL/min. Whole-cell recordings were made using electrodes with resistances between 3 and 7 MΩ. Electrodes were fabricated from 4” thin-walled glass capillaries (1.5 OD/1.12 ID; World Precision Instruments) using a Flaming/Brown micropipette puller (P-97; Sutter Instruments). Recording electrodes were filled with an intracellular solution composed of (in mM): 135 KMeSO_4_, 12 NaCl, 10 HEPES, 0.5 EGTA, 2 Mg^2+^-ATP, and 0.3 Tris-GTP. All chemical components were obtained from either Sigma‒Aldrich or Fisher Scientific.

### Timing of the Estrous Cycle

Vaginal samples were collected from all female mice immediately following decapitation. A micropipette tip was inserted 1–2 mm into the vaginal opening and 10 μL of 0.9% saline was flushed into the vagina three times. The resulting sample was then applied to a glass slide and a smear was made. Each smear was allowed to dry fully before staining with Wright-Giemsa stain. Under 20X magnification, each smear was analyzed for the presence of nucleated epithelial cells, anucleated epithelial cells, and leukocytes and was classified as representing proestrus, estrus, metestrus, or diestrus as described by Cora and colleagues.(32)

### Data Acquisition

Whole-cell recordings were collected from D1R + MSNs located in the NAcSh (Fig. 1b). Cells were identified as D1R + by the presence of epifluorescent illumination of tdTomato using the MRK200 Modular Imaging system (Siskiyou Corporation). Data was acquired utilizing a CV203BU headstage mounted on a vibration isolation table and an Axopatch 200B amplifier, with 1 kHz filtering. Data was digitized at 5 kHz through a Digidata 1440A interface board using Clampex 10.3 (all products by Molecular Devices, Sunnyvale, CA, United States). Immediately after obtaining whole-cell configuration, cells were selected for further experimentation by exhibiting a series resistance of less than 33 MΩ and having a resting membrane potential of less than or equal to −60 mV. Any electrophysiology recording during which series resistance changed by more than 20% or exceeded 33 MΩ was excluded from statistical analysis. Membrane voltage responses were measured by recording 20 sweeps 750 ms in duration that included in order: 50 ms of no current application, 150 ms of −20 pA, 150 ms of no current application, 300 ms of either a hyperpolarizing or depolarizing current, and 100 ms of no current application. Hyperpolarizing or depolarizing current steps increased at a rate of 50 pA from − 400 pA to 550 pA. The resting membrane potential was defined as the average voltage (mV) during the initial 50 ms of the 0 pA sweep. The steady state of the voltage response to the − 20 pA step was used to calculate the input resistance. Rheobase was defined as the amplitude of the current step that elicited the first action potential (AP) for each cell. AP waveforms were analyzed using the first AP in the rheobase step, unless the AP fired too close to the end of the step to measure AHPs; in these cases, the first AP of the next step was used. The AP threshold was defined as the point at which dV/dt exceeded 10 mV/ms for the first AP. AP amplitude was calculated by subtracting the AP threshold from the AP peak voltage. AP half-width was defined as the duration between the AP threshold and the AP half-amplitude. AP after-hyperpolarization potential (AHP) amplitudes were defined as the difference between the threshold voltage and the most negative voltage within 5 ms of threshold, and the voltages at 10 and 15 ms after the AP threshold, for fast (fAHP), medium (mAHP) and slow (sAHP), respectively.(33, 34) The sweep with the maximum number of APs was used to determine the spike frequency adaptation ratio (SFA) and to determine the maximum peak-to-peak frequency using the shortest interval between two APs. SFA was calculated by dividing the interval between the first two APs of the sweep by the interval between the last two APs of the sweep. To monitor spontaneous excitatory postsynaptic currents (sEPSCs), the membrane potential was clamped at −80 mV. Raw data analysis was performed in Clampfit 10.6 (Molecular Devices). Data was acquired blind to the stage of the Estrous cycle. Data analysis took place after all data was collected and was performed blind to sex and stage of Estrous. The sEPSC frequency and average amplitude were determined over a 1–3-minute period by utilizing the Clampfit template search feature and rejecting events smaller than 5 pA.

### Statistics

Statistical analyses were performed in GraphPad Prism 9.3. The current step-evoked action potential number was analyzed by two-way ANOVA, with sex or stage of Estrous as the between-group factor and the current step as the repeated measure. When comparing sex, variables were analyzed using unpaired Student’s *t* tests; Welch’s correction was applied when homogeneity of variance was violated. When comparing the stages of Estrous, variables were analyzed using a one-way ANOVA or Kruskal‒Wallis test when data were both nonnormally distributed and in violation of homogeneity of variance. Depiction of variance on figures was specific to allow for better visualization. Table 1 shows details of the statistical analysis.

## Results

**Figure 2:**
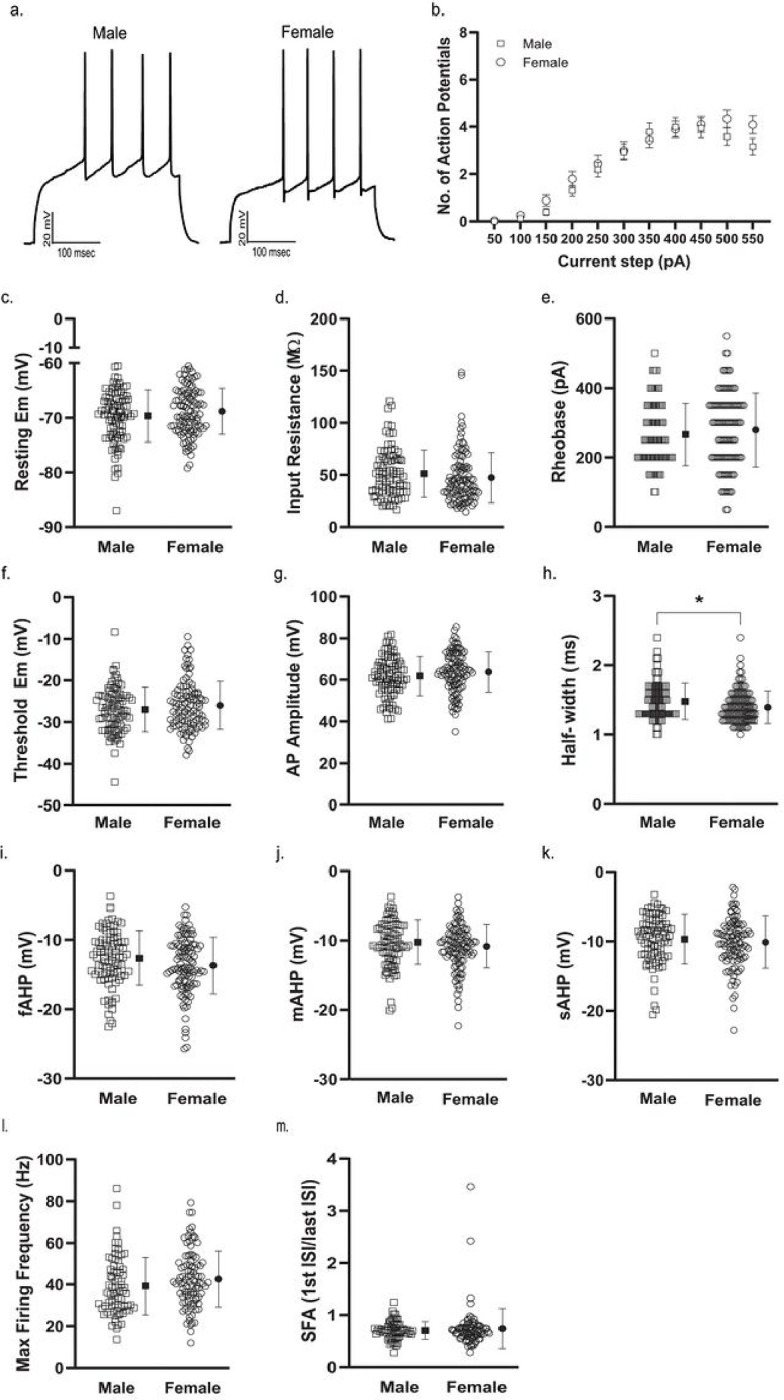
Membrane properties and cellular excitability of D1R+ MSNs from male and female Drd1a-tdTomato mice. ***a.*** Example membrane voltage traces showing responses to a 400 pA current step for D1R+ MSNs from male and female mice. ***b.*** Number of action potentials (APs) elicited over increasing depolarizing current steps (50 pA – 550 pA, 300 ms in duration) in cells from male (open squares) and female (open circles; n = 111/43) mice. Error bars indicate thestandard error of the mean. For b-d, n (cells/mice) = 91/39 male (squares), 111/43 female (circles). ***c.*** Resting membrane potential (Em) of D1R+ MSNs from male and female mice. For c-m, individual values for each neuron (open symbols) are plotted and are presented alongside the group mean (closed symbols) and standard deviation. ***d.*** Input resistance. ***e***. AP rheobase. For e-k, n (cells/mice) = 90/38 male (squares), 108/43 female (circles). ***f.*** AP threshold. ***g.*** AP amplitude. ***h.*** AP half-width was significantly longer in duration in D1R+ MSNs from male mice (squares; n = 90/38) than from female (circles; n = 108/43) mice [*p = 0.0188, unpaired t test]. ***i.*** Fast after-hyperpolarizing amplitude (fAHP) following the first elicited AP. ***j.*** Medium after-hyperpolarizing amplitude (mAHP) following the first elicited AP. ***k.*** Slow after-hyperpolarizing amplitude (sAHP) following the first elicited AP. ***l.*** Maximum peak-to-peak firing frequency of APs from male (squares; n = 76/36) and female (circles; n = 97/42) mice. ***m.*** Spike frequency adaptation (SFA) during the current step that elicited the maximum number of APs in male (squares; n = 63/34) and female (circles; n = 86/38) mice.

**Figure 3:**
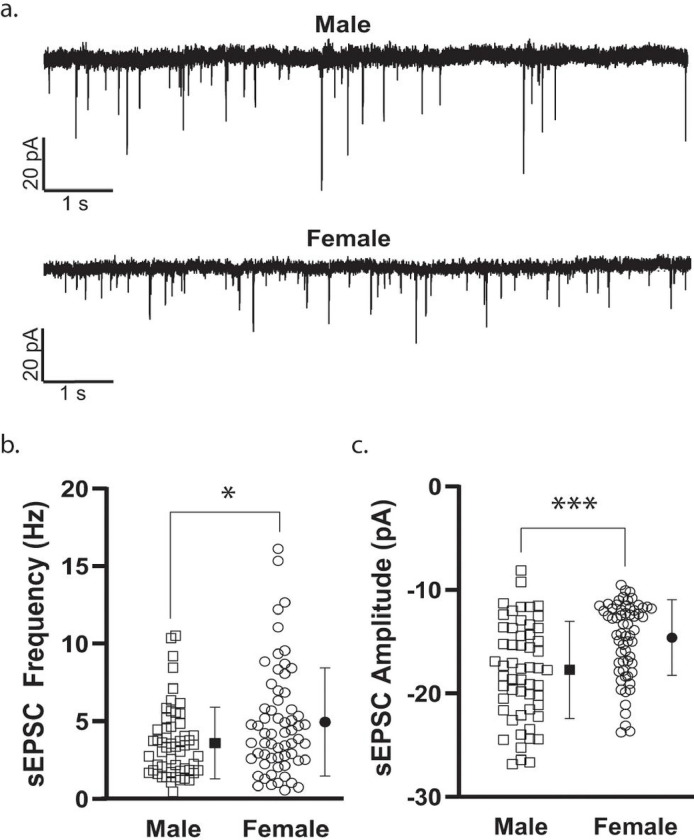
Sex differences in spontaneous D1R+ MSN glutamatergic transmission. ***a.*** Example traces of spontaneous excitatory postsynaptic currents (sEPSCs) from male and female mice. ***b.*** D1R+ MSNs from male mice exhibited areduced frequency of sEPSCs compared to female mice [*p = 0.0134, unpaired t test with Welch’s correction]. For b and c, individual values for each neuron (open symbols) are plotted and are presented alongside the group mean (closed symbols) and standard deviation. n (cells/mice) = 55/33 male (squares), 62/33 female (circles). ***c.*** Spontaneous event amplitude was increased in D1R+ MSNs from male mice compared to female mice [***p = 0.0001, unpaired t test].

To establish whether measures of membrane properties and cellular excitability in NAcSh D1R + MSNs during mid-adolescence differed by sex, we collected whole-cell electrophysiology data in brain slices from male (n = 39) and female (n = 43) mice. We found no significant effect of sex on the number of action potentials (APs) elicited over increasing depolarizing current steps (F(10, 2000) = [1.077], *p* = 0.3759)^b^, resting membrane potential (*t*(200) = 1.316, *p* = 0.1895)^c^, input resistance (*t*(200) = 1.145, *p* = 0.2537)^d^, rheobase (*t*(196) = 0.9219, *p* = 0.3577)^e^, threshold (*t*(196) = 1.243, *p* = 0.2155)^f^, and AP amplitude (*t*(196) = 1.414, *p* = 0.1591)^g^ (Fig. 2a–g). When examining AP duration, as measured by AP half-width, we found APs to be significantly longer in duration in cells from male mice compared to those observed in cells from female mice (*t*(196) = 2.368, *p* = 0.0188, η² = 0.0278)^h^ (Fig. 2h). All other measures were found to be similar, with no sex difference observed for fAHP (*t*(196) = 1.822, *p* = 0.0699)^i^, mAHP (*t*(196) = 1.325, *p* = 0.1867)^j^, sAHP (*t*(196) = 0.8012, *p* = 0.4240)^k^, maximum peak-to-peak frequency of AP firing (*t*(171) = 1.626, *p* = 0.1057)^l^, and SFA (Welch’s *t*(124.2) = 0.7793, *p* = 0.4373)^m^ (Fig. 2i–m). When examining spontaneous glutamatergic transmission, we found that D1R + MSNs from female mice exhibited a significantly higher frequency of excitatory events (Welch’s *t*(106.8) = 2.515, *p* = 0.0134, η² = 0.0559)^n^ and a smaller average event amplitude (*t*(115) = 4.013, *p* = 0.0001, η² = 0.1228)^o^ than was measured in cells from male mice (Fig. 3).

**Figure 4:**
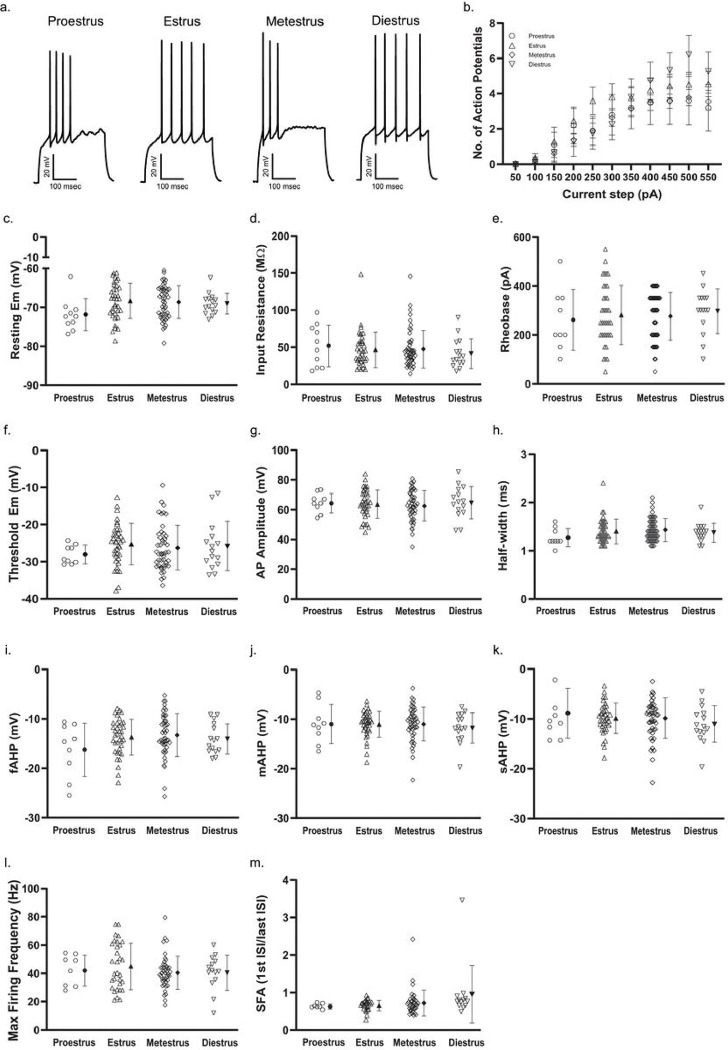
D1R+ MSN membrane properties and excitability in females across the four stages of the Estrous cycle, proestrus, estrus, metestrus and diestrus. ***a.*** Example membrane voltage traces showing responses to a 400 pA current step. ***b.*** Number of action potentials (APs) elicited over increasing depolarizing current steps (50 pA – 550 pA, 300 ms in duration). Error bars indicate the standard error of the mean. For b-d, n (cells/mice) = 10/4 during proestrus (hexagons), 37/15 during estrus (triangles), 49/16 during metestrus (diamonds), and 15/8 during diestrus (inverted triangles). ***c.*** Resting membrane potential (Em). For c-m, individual values for each neuron (open symbols) are plotted and are presented alongside the group mean (closed symbols) and standard deviation. ***d.*** Input resistance. ***e.*** AP rheobase. For e-k, n (cells/mice) = 9/4 during proestrus (hexagons), 37/15 during estrus (triangles), 47/16 during metestrus (diamonds), and 15/8 during diestrus (inverted triangles). ***f.*** AP threshold. ***g.*** AP amplitude. ***h.*** AP half-width. ***i.*** Fast after-hyperpolarizing amplitude (fAHP) following the first elicited AP. ***j.*** Medium after-hyperpolarizing amplitude (mAHP) following the first elicited AP. ***k.*** Slow after-hyperpolarizing amplitude (sAHP) following the first elicited AP. ***l.*** Maximum firing frequency of APs in response to a depolarizing current step during proestrus (open hexagons; n = 8/4), estrus (open triangles; n = 31/14, metestrus (open diamonds; n = 43/16) and diestrus (open inverted triangles; n = 14/8). ***m***. Spike frequency adaptation (SFA) during the current step that elicited the maximum number of APs during proestrus (open hexagons; n = 7/4), estrus (open triangles; n = 28/12), metestrus (open diamonds; n = 37/14) and diestrus (open inverted triangles; n = 13/8).

**Figure 5:**
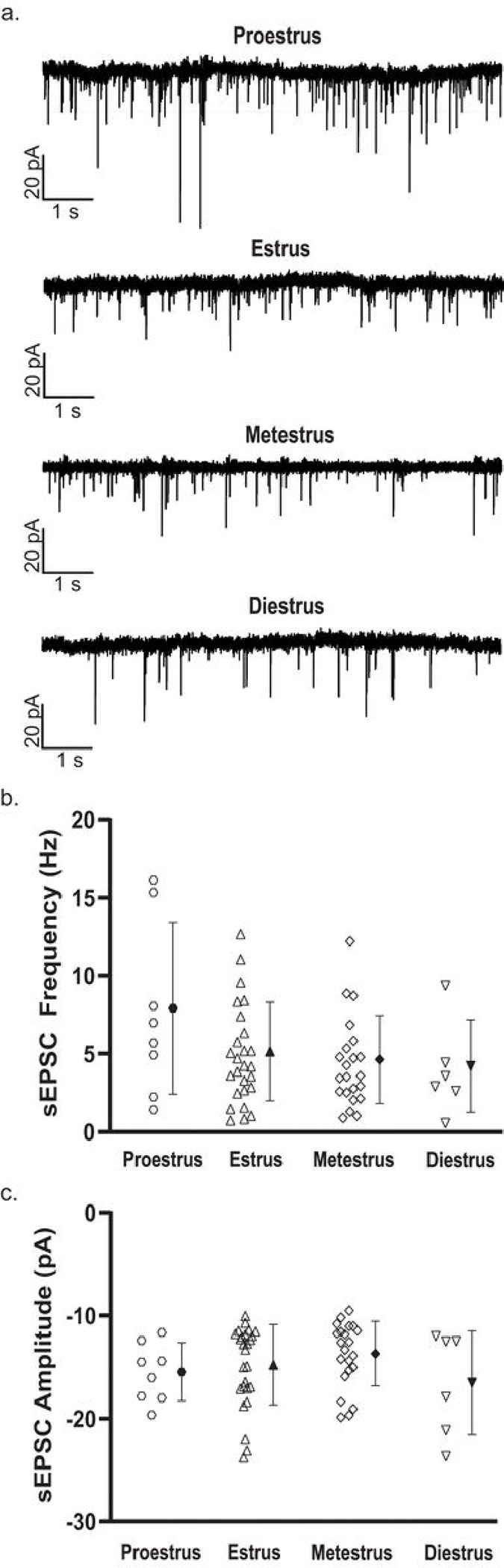
Spontaneous glutamatergic transmission in D1R+ MSNs from females across the stages of the Estrous cycle. ***a.*** Example traces of whole-cell sEPSCs for females in proestrus, estrus, metestrus, and diestrus. ***b.*** The frequency of spontaneous excitatory events was similar across the stages of Estrous. For b & c, individual values for each neuron (open symbols) are plotted and are presented alongside the group mean (closed symbols) and standard deviation. n (cells/mice) = 8/4 during proestrus (hexagons), 26/12 during estrus (triangles), 22/13 during metestrus (diamonds), 6/4 during diestrus (inverted triangles). ***c.*** Spontaneous event amplitude was similar across the stages of the Estrous cycle.

We then wished to investigate whether the sex differences we observed could be attributed to a stage or stages of the Estrous cycle and grouped cells from female mice into proestrus, estrus, metestrus, or diestrus for comparison. We observed no significant difference between stages of Estrous for any measure of D1R + MSN membrane properties nor cellular excitability, including: resting membrane potential (F(3, 107) = [2.006], *p* = 01175)^p^, input resistance (F(3, 107) = [0.4046], *p* = 0.7500)^q^, number of APs elicited over increase depolarizing current steps (F(30, 1070) = [0.8695], *p* = 0.6697)^r^, rheobase (F(3, 104) = [0.2369], *p* = 0.8705)^s^, AP threshold (F(3, 104) = [0.6072], *p* = 0.6118)^t^, AP amplitude (F(3, 104) = [0.2261], *p* = 0.8781)^u^, AP half-width (F(3, 104) = [1.257], *p* = 0.2932)^v^, fAHP (F(3, 104) = [1.396], *p* = 0.2484)^w^, mAHP (F(3, 104) = [0.2528], *p* = 0.8592)^x^, sAHP (F(3, 104) = [0.6739], *p* = 0.5699)^y^, maximum peak-to-peak frequency of AP firing (H(3) = [1.052], *p* = 0.7886)^z^, and SFA (H(3) = [5.606], *p* = 0.1324)^aa^ (Fig. 4). Additionally, spontaneous glutamatergic transmission did not differ by stage of Estrous, as no significant difference was observed for the frequency of spontaneous excitatory events (F(3, 58) = [2.064], *p* = 0.1149)^ab^ or for average event amplitude (F(3, 58) = [1.177], p = 0.3262)^ac^ (Fig. 5).

## Discussion

Our results indicate that D1R + MSNs in the NAcSh differ by sex in action potential (AP) waveform and spontaneous glutamatergic transmission. In summary, we found AP duration to be longer in males and that females exhibited a lower amplitude but increased frequency of sEPSCs. There was no evidence that any one stage of the Estrous cycle drove the observed sex differences or a lack thereof. To our knowledge, this is the first body of work to focus on D1R + MSNs in the NAcSh during mid-adolescence in pubertal aged mice.

Here, we observed a longer AP duration in D1R + MSNs from male mice relative to females. The width of the mammalian action potential waveform is influenced by the activation of both voltage-gated sodium (Na_v_) and potassium (K_v_) channels.(35) While blocking Na_v_ channels has previously been shown to lengthen AP duration in MSNs in the NAc of adolescent rats, this manipulation also resulted in a reduction in AP amplitude.(36) As we found no evidence of sex differences in AP amplitude, our results suggest that rather than Na_v_ channels underlying the observed sex difference, K_v_ channels may be responsible. Activation of K_v_ channels limits cellular excitability by repolarizing the membrane after Na_v_ channels close.(37) Although there are many subunits of K_v_ channels expressed in the striatum, recent findings from Otuyemi et al. (38) indicate that, at least in the dorsal striatum, D1R + MSNs from adult mice of both sexes express K_v_2.1 and K_v_4.2 channels. The localization of K_v_2.1 (distributed across the soma and proximal dendrites) suggests that these channels may have contributed to our observed sex difference in AP waveform, rather than K_v_4.2 channels (which are on distal dendrites). K_v_2.1 channels may be found in non-conducting clusters or in conducting non-clusters, and in response to glutamate, clustered K_v_2.1 channels can disperse across the cell surface.(38–41) Therefore, it is plausible that during mid-adolescence, the number of and/or clustering patterns of K_v_2.1 changes over murine ontogeny, possibly in response to glutamatergic transmission, and that the ontogeny of K_v_2.1 is impacted by sex but not by the stage of the Estrous cycle. Further evidence supporting this notion can be found in the work of Brundage and colleagues (42), who also observed evidence for sex differences in the function of K_v_ channels in the striatum of mice from PND 30 onward.

Spontaneous excitatory postsynaptic currents (sEPSCs) differed in both frequency and amplitude in a sex-dependent manner. Here, we observed D1R + MSNs in the NAcSh of female mice to have a decreased event amplitude and an increased event frequency when compared to the same cell type in males. As cells were held at −80 mV during these electrophysiology recordings, N-methyl-D-aspartate (NMDA)-type ionotropic glutamate receptors can be eliminated as strong contributors to this sex difference due to the magnesium block at this voltage.(43) Kainate receptors (KAR) GluR6, GluR7 and KA2 are expressed in the striatum of mice and rats,(44–46) and as we did not pharmacologically isolate AMPARs here, we cannot exclude KAR involvement in the sex differences observed for sEPSC frequency or amplitude. However, when considering that the majority of KARs are expressed outside of the postsynaptic density,(47) their involvement as a major contributor to sex differences in EPSCs appears unlikely, although to our knowledge, KAR distribution has not yet been investigated during mid-adolescence in both sexes. Thus, α-hydroxy-5-methyl-4-isoxazolepropionic acid type ionotropic glutamate receptors (AMPARs) are likely the predominant mediators of the observed sex differences in excitatory synaptic transmission.

Functional AMPARs are present on mature synapses, function in basal neurotransmission, and have been shown in other brain regions to have a high likelihood of being present on large dendritic spines.(48–52) Previous work by Forlano and Woolley (53) suggests that female MSNs in the NAc exhibit a greater dendritic spine density and a greater number of large-headed spines than males. Furthermore, during adolescence, synaptic pruning of D1R + and D2R + MSNs has been observed to be greater in males.(1) Thus, it is plausible that the increased SEPSC frequency observed in females is a reflection of having a greater number of functional synapses than males. In addition to synapse number, overall network activity influences sEPSC frequency. Furthermore, differences in frequency can be mediated by both pre- and postsynaptic mechanisms, such as altered presynaptic release probability or insertion of AMPARs into previously “silent” synapses, respectively.(54) The latter phenomenon, through which postsynaptic AMPAR insertion results in increased EPSC event frequency, is a well-documented developmental event. (54) This again points toward differences in the number of functional synapses as a plausible explanation for the increased event frequency in females.

Our findings do not elucidate what specific alteration or combination of changes in AMPARs contributed to the sex difference in amplitude observed here. Changes in the number of AMPARs expressed on the cell surface, incorporation of GluA2 subunits, alternative splicing of the flip-flop module, and editing at the R/G site can influence the amplitude of postsynaptic currents by impacting desensitization, recovery from desensitization, and single channel conductance.(55–62) There is also evidence that the expression, editing, and alternative splicing of AMPARs changes over ontogeny in both mice and rats.(59, 63, 64) For example, editing at the Q/R site, which reduces single channel conductance, is completed prior to parturition in mice.(65, 66) Thus, our findings highlight potential sex differences in AMPAR ontogeny.

To our knowledge, only Willett and colleagues (27) have reported on excitatory synaptic transmission and membrane properties of NAcSh MSNs from both sexes, finding no evidence of significant sex differences. Their work was conducted in rats at PND 21 in MSNs not identified by subtype, and the recorded neurons were in an area of the NAcSh that was more dorsal to that in our study. Thus, one interpretation of our finding of significant sex differences in the NAcSh during PND 35–47, while Willett and colleagues did not find any at PND 21, could be that sex differences in glutamate transmission and action potential half-width emerge between pre- and mid-adolescence. On the other hand, the discrepancy in findings might also be a result of important methodological differences. Therefore, to better understand exactly when and where sex differences emerge, future studies should target specific populations of MSNs at multiple developmental stages.

## Perspectives and Significance

Using mid-adolescent, pubertal aged, gonadally intact mice, we explored potential sex-based variations in membrane properties and excitatory synaptic transmission among dopamine D1 receptor-expressing medium spiny neurons in the nucleus accumbens shell. Additionally, we assessed potential impacts of Estrous cycle stage on these measures, in females. Our findings suggest potential sex differences in potassium channel regulation of D1 receptor-expressing medium spiny neuron waveforms and potential sex differences in influence(s) of AMPAR number, composition, activation, and/or distribution on glutamatergic transmission during mid-adolescence. Notably, these distinctions were not attributed to any specific stage of the Estrous cycle, suggesting a baseline divergence in the neural structure and/or function between male and female mice during mid-adolescence. Our results underscore the complexity of neural processes during adolescence and highlight the significance of sex-specific considerations in the functioning of the shell of the nucleus accumbens. Future research is essential to pinpoint the precise molecular mechanisms involved, offering potential targets for future studies investigating disorders or conditions with prevalence and symptomology that differ by sex.

## Data Availability

The datasets used and analyzed during the current study are available from the corresponding author on reasonable request.

## Highlights

- Sex, as a biological variable, impacts measures of membrane properties and basal glutamate transmission of dopamine D1 receptor-expressing medium spiny neurons within the shell of the nucleus accumbens during mid-adolescence.
- The stage of the Estrous cycle does not appear to drive the observed sex differences in membrane properties or basal glutamate transmission.
- Within females, no membrane properties or measures of glutamate transmission differed between stages of the Estrous cycle.
